# Frequent use of selected sugary products associates with thinness, but not overweight during preadolescence: a cross-sectional study

**DOI:** 10.1017/S0007114520001361

**Published:** 2020-06-18

**Authors:** Sohvi Lommi, Rejane Augusta de Oliveira Figueiredo, Hely Tuorila, Heli Viljakainen

**Affiliations:** 1 Department of Public Health, University of Helsinki, 00014 Helsinki, Finland; 2 Folkhälsan Research Center, 00250 Helsinki, Finland; 3 Faculty of Medicine, University of Helsinki, 00014 Helsinki, Finland; 4 Department of Food and Nutrition, University of Helsinki, 00014 Helsinki, Finland

**Keywords:** Consumption frequency, Sugary products, BMI, Childhood, Adolescence

## Abstract

Convincing evidence suggests that diets laden with added sugar, specifically sugar-sweetened beverages, associate with excess weight in children. The relationships between sugar consumption frequency and BMI remain less well studied. We, therefore, evaluated children’s consumption frequency of selected sugary products (*n* 8461; mean age 11·1 (sd 0·9) years) selected from the Finnish Health in Teens cohort study. Using a sixteen-item FFQ including six sugary products (chocolate/sweets, biscuits/cookies, ice cream, sweet pastry, sugary juice drinks and sugary soft drinks), we calculated a Sweet Treat Index (STI) for the frequency of weekly sugary product consumption and categorised children based on quartiles (Q) into low (Q1, cut-off < 4·0), medium (Q2 + Q3, range 4·0–10·5) and high STI (Q4, cut-off > 10·5), and as thin, normal and overweight/obese based on the measured BMI. Through multinomial logistic regression analyses, we found that subjects with a high STI exhibited a higher risk of being thin (OR 1·20, 95 % CI 1·02, 1·41) and lower risk of being overweight (OR 0·79, 95 % CI 0·67, 0·92), while subjects with a low STI were at higher risk of being overweight (OR 1·32, 95 % CI 1·14, 1·53). High consumption frequencies of salty snacks, pizza and hamburgers most closely were associated with a high STI. Our findings suggest that consuming sugary products at a high frequency does not associate with being overweight. The relationship between a low consumption frequency and being overweight suggests that overweight children’s consumption frequency of sugary products may be controlled, restricted or underreported.

Even though mean BMI appears to have recently levelled off in some developed countries^(1)^, childhood obesity remains a major concern given its association with several health risks, such as high blood pressure and type 2 diabetes, and is likely to continue into adulthood^(2,3)^. The causes of obesity remain complex and multifactorial^(4)^. In recent decades, it has been suggested that sugar may play an important role in the obesity epidemic: that is, the prevalence of obesity has increased simultaneously with sugar consumption^(5)^. Yet, the issue remains controversial as recent data suggest that sugar intake is either stable or decreasing worldwide^(6)^. Nonetheless, diets featuring large amounts of sugary foods and drinks predict weight gain, and the intake of sugars and sugar-sweetened beverages associates with BMI in adults, while in children, such associations are primarily limited to sugar-sweetened beverages^(7)^.

One of the main dietary challenges among children and adolescents in many developed countries, including Finland, lies in the high consumption of sucrose-rich drinks and snacks^(8–10)^. According to estimates, beverages and food consumed as snacks provide as much as 42 % of daily energy intake and two-thirds of the daily sucrose intake in Finnish children^(11)^. Excess sugar deteriorates dietary quality^(12,13)^ and may imperceptibly lead to a positive energy balance. Sugar-sweetened beverages specifically may prove problematic, since energy in liquid form may not provide similar satiety-promoting effects as energy consumed from solid sources^(14)^. The WHO recommends that the intake of free sugars (i.e. mono- and disaccharides added to foods, and sugars naturally present in honey, syrups and fruit juices) should not exceed 10 % of the total energy intake^(15)^.

No specific recommendations exist defining the frequency of consumption of sugary products when considering health outcomes beyond dental health. To minimise the risk of caries, the 2012 Nordic Nutrition Recommendations advise decreasing the consumption frequency of sugar-containing foods and drinks^(16)^, while WHO recommends restricting such consumption to no more than four times daily^(17)^. As a part of efforts to improve children’s oral health and following Sweden’s example, the Finnish Dental Association recommended restricting eating sweets to 1 d/week – designated ‘a candy day’ – already in the 1970s^(18,19)^. Associations between the consumption frequency of sweet products and body weight remain less well studied, however. A meta-analysis indicated that children and adolescents in the highest consumption group, compared with reference consumption, exhibited a lower risk for being overweight^(20)^. However, this meta-analysis combined studies assessing both consumption frequencies and the intake of individual food items. Thus, further evidence of the consumption frequency of sugary products and its association with BMI is needed in order to understand the behavioural dimensions and patterns of sweet foods and drinks consumption and health outcomes. This study, therefore, aimed to assess the relationship between weight status and the frequency of selected sugary product consumption among nearly 9000 Finnish preadolescents. The selected energy-dense and nutrient-poor products are generally consumed as sweet treats. Furthermore, we aimed to understand the relationship between sugary product consumption and the consumption of some non-sweet foods alongside lifestyle factors such as sleep and physical activity (PA).

## Methods

### Subjects and data collection

This cross-sectional study derives data from the Finnish Health in Teens cohort, which includes approximately 11 400 Finnish children aged 9–12 years. Data were collected during 2013 and 2014 in schools across Finland. Municipalities with high population density and areas surrounding them were selected. In total, 496 (95 %) schools agreed to participate. In schools, all children in grades 3–6 were invited to the study. The response rate was 36 % without sending reminders to the invitees. The study protocol has been described in detail elsewhere^(21)^. Here, we selected 8461 children with information available on fifteen food items from a FFQ, age, sex, BMI, maternal socio-economic status (SES), sleep duration and PA. The research was conducted according to the Declaration of Helsinki, and children and one parent per child provided their written informed consent. The Ethics Committee of the Hospital District of Helsinki and Uusimaa approved the study protocol (169/13/03/00/10).

### Anthropometric data and questionnaires

Subjects’ weight, height and waist circumference were measured in a standardised way by trained fieldworkers with equipment calibrated daily^(21)^. BMI was calculated as weight (kg)/height squared (m^2^), and subjects were classified as thin (grade 1, 2 and 3 thinness), normal- or overweight or obese according to age- and sex-specific cut-offs based on the International Obesity Task Force suggestions^(22)^. Because of the low number of obese subjects in the sample (*n* 208, 2·5 %) and therefore, the lack of statistical power in the multinomial logistic regression analysis, overweight and obese subjects were combined into a single category (hereafter, referred to as overweight). Subjects answered a short FFQ and questions related to sleep and PA. The self-administered FFQ included sixteen items, covering the overall diet and comprising foods typically eaten by young people in Finnish culture. Six items represented sugary products, nine other foods and the final item was water (nonnutritious, excluded from the present analysis). The consumption frequency of each item during the preceding month was rated on a seven-point scale, consisting of ‘not at all’, ‘less than once a week’, ‘once a week’, ‘2–4 times a week’, ‘5–6 times a week’, ‘once a day’ and ‘several times a day’. Our FFQ was adapted from the FFQ used in the WHO’s Health Behaviour in School-Aged Children Study (HBSC), conducted in several countries around the world, including Finland. The HBSC FFQ has been validated against a 7-d food diary and a 24-h food behaviour checklist and retested in Belgium and Italy among 11–12-year-old children, showing acceptable validity and reliability^(23,24)^. In addition, the International Study of Childhood Obesity, Lifestyle and the Environment used a twenty-three-item FFQ comprising similar food items as ours, and it has been validated against a 3-d pre-coded food diary and retested in three countries, Finland included, confirming validity and reliability^(25)^. Moreover, in the HBSC study, the validity of an index combining sweets and soft drinks was tested, which showed good test–retest reliability (0·82) and moderate correlation (0·53) with the food diary^(24)^.

Sleep duration and PA likely modify the association between the consumption of sugary products and BMI. Thus, we included them in the analysis. Subjects were asked about their bed and wake-up times on weekdays and weekends, and sleep durations during weekdays and weekends were computed separately. We chose to carry out our analysis using sleep duration during weekdays, since sleep during the week appears shorter in duration and associates more strongly with food choices and being overweight than weekend sleep duration^(26,27)^. We categorised children into three groups according to age-specific recommendations: those who sleep the recommended amount of time, those who sleep less than recommended and those who sleep more than recommended^(28)^. The recommended amount of sleep in a 24-h period is 9–12 h for 6–12-year-old children.

In addition, we assessed the level of PA by asking the subjects how many hours a week they exercise during their leisure time. We then categorised them into two groups: those who exercise 7 h or more and those who exercise <7 h. We chose the cut-off of 7 h based on the recommendation of at least 60 min of daily moderate- or vigorous-intensity PA for school-aged children and adolescents^(29)^. Any exercise during leisure time, not including exercise at school or on the way to/from school, was considered PA. Data related to sex and age were obtained from questionnaires or the informed consent form. The occupation of the mother at the time of the child’s birth, used as the indicator of maternal SES, was received from the Medical Birth Register from the National Institute for Health and Welfare (THL)^(30)^. The SES groups in the register are: upper-level employees, lower-level employees, manual workers, students, self-employed persons, stay-at-home mothers and others including unemployed and pensioners. Due to small group sizes, we regrouped self-employed persons, stay-at-home mothers and others into one category (named ‘other’).

### Sweet Treat Index

We calculated a sum variable Sweet Treat Index (STI) to represent the weekly frequency of selected sugary product consumption. Six foods and drinks represented these products: (1) chocolate and sweets; (2) biscuits and cookies; (3) sweet pastry; (4) ice cream; (5) sugary juice drinks and (6) sugary soft drinks. These were selected as sugary drinks, milk products, chocolate and confectionery are common sources of sucrose in Finnish children’s and adolescents’ diets^(8,11,31)^. Moreover, sweet pastry, cookies, ice cream, sweets and chocolate are consumed 1–2 times/week among secondary school pupils^(32)^. Subjects’ item ratings were further recoded into times/week to better illustrate the weekly consumption frequency: ‘not at all’ as 0, ‘less than once a week’ as 0·5 (we assumed an average consumption of twice per month), ‘once a week’ as 1, ‘2–4 times a week’ as 3, ‘5–6 times a week’ as 5·5, ‘once a day’ as 7 and ‘several times a day’ as 14 times a week (we assumed an average consumption of twice daily). A similar scoring was previously used^(26)^. The total index was based on the sum frequency of the weekly sugary food consumption of the six items, ranging from 0 to 84. The higher the score, the greater the frequency of consumption. Based on the index, we categorised the children into quartiles (Q) to compare subjects with low and high frequencies. The second and third quartiles were further combined, and groups were labelled as low (Q1, cut-off < 4·0), medium (Q2 + Q3, range 4·0–10·5) and high STI (Q4, cut-off > 10·5). We calculated Spearman’s correlations for STI and individual sugary food items and yielded a direct correlation coefficient of >0·5 for each item (Table 1), indicating that all items contributed to STI. The internal consistency was tested using Cronbach’s α coefficient, resulting in a value of 0·73, which is considered good^(33)^. Removing any of the six items would have reduced Cronbach’s α coefficient, indicating that all items should remain in the index.

**Table 1.** Spearman coefficients of the correlation between the Sweet Treat Index (STI) and individual food items included in the index

	STI
Food items	Spearman’s coefficient
Chocolate and sweets	0·550
Sweet pastry	0·556
Biscuits/cookies	0·652
Ice cream	0·514
Sugary juice drinks	0·683
Sugary soft drinks	0·560

### Statistical methods

Categorical variables were compared using the *χ*^2^ test. To evaluate associations between STI groups and BMI groups, we calculated OR and 95 % CI using the multinomial logistic regression, comparing thin and overweight children with normal-weight children. We used medium STI as the reference category. The multinomial logistic regression models were adjusted for sex, age, maternal SES and PA and stratified based on sleep duration during weekdays. The interactions between STI and sleep and STI and PA, respectively, were tested using the likelihood ratio test.

Statistical analyses were performed using IBM’s SPSS version 22 (IBM) and SAS version 9.4 (SAS Institute Inc.). We set the level of statistical significance to *P* < 0·05.

## Results

Among all 8461 subjects, 52·5 % (*n* 4444) were girls and 47·5 % were boys (*n* 4017), with a mean age of 11·1 (sd 0·9) years. Overall, 11·5 % of the children were thin, 73·9 % were normal weight and 14·7 % were overweight. The average frequency of sugary product consumption was 8·7 (sd 7·6) times/week, ranging from 0 to 84 times/week. Table 2 shows the characteristics of subjects by BMI groups. The distribution of boys and girls differed according to BMI: there were a higher proportion of girls in the thin group compared with overweight group, while for boys, the opposite was observed. Maternal SES differed between BMI groups: manual workers were over-represented and upper-level employees were under-represented in the overweight group. The proportion of subjects who slept less than recommended was highest in the overweight group, as was the proportion of subjects reporting <7 h of PA a week.

**Table 2.** Children’s characteristics according to BMI categories (*n* 8461)* (Numbers and percentages)

	BMI								
	Thin		Normal weight		Overweight				*χ*^2^, *P*
	*n*	%	*n*	%	*n*	%	Total *n*	%	
Sex									
Girl	581	60·0	3224	51·6	639	51·4	4444	52·5	<0·001
Boy	388	40·0	3026	48·4	603	48·6	4017	47·5	
Maternal socio-economic status									
Upper-level employees	282	29·1	1961	31·4	313	25·2	2556	30·2	<0·001
Lower-level employees	398	41·1	2427	38·8	509	41·0	3334	39·4	
Manual workers	100	10·3	680	10·9	209	16·8	989	11·7	
Students	120	12·4	685	11·0	117	9·4	922	10·9	
Other	69	7·1	497	8·0	94	7·6	660	7·8	
Physical activity per week									
<7 h	474	48·9	2646	42·3	661	53·2	3781	44·7	<0·001
≥7 h	495	51·1	3604	57·7	581	46·8	4680	55·3	
Sleep duration during weekdays									
<Recommended	52	5·4	491	7·9	146	11·8	689	8·1	<0·001
Recommended	897	92·6	5640	90·2	1069	86·1	7607	89·9	
>Recommended	20	2·1	119	1·9	27	2·2	166	2·0	

* The group ‘overweight’ includes obese subjects.

Table 3 provides the distributions for the maternal SES, sleep duration and PA, and for the consumption frequencies of other food items in low, medium and high STI groups. Maternal SES differed by the STI groups: upper-level employees were more common in the low STI group than in the high STI group, while an opposite tendency was observed among lower-level employees and manual workers. The highest consumption frequencies of ‘hamburgers or hot dogs’, ‘pizza’ and ‘salty snacks’ were found in the high STI group, and conversely, the lowest consumption rates of these items were found in the low STI group. We observed a similar trend for ‘fresh juice’. A high consumption of ‘fresh vegetables’ was more common among subjects with a low STI than those with a high STI. The differences in the consumption of ‘dark bread’, ‘cooked vegetables’ and ‘fruits or berries’ were less pronounced, although statistically significant.

**Table 3.** Distribution of lifestyle factors and food item frequencies by Sweet Treat Index (STI) groups (*n* 8461) (Numbers and percentages)

	STI						Total *n*	%	
	Low		Medium		High				*χ*^2^, *P*
	*n*	%	*n*	%	*n*	%			
Lifestyle factors									
Maternal socio-economic status									
Upper-level employees	643	34·1	1375	30·2	538	26·5	2556	30·2	<0·001
Lower-level employees	686	36·4	1786	39·3	862	42·4	3334	39·4	
Manual workers	195	10·4	533	11·7	261	12·8	989	11·7	
Students	232	12·3	492	10·8	198	9·7	922	10·9	
Other	127	6·7	360	7·9	173	8·5	660	7·8	
Sleep duration during weekdays									
<Recommended	115	6·1	328	7·2	246	12·1	689	8·1	<0·001
Recommended	1726	91·7	4129	90·8	1751	86·2	7606	89·9	
>Recommended	42	2·2	89	2·0	35	1·7	166	2·0	
Physical activity per week									
<7 h	805	42·8	1978	43·5	998	49·1	3781	44·7	<0·001
≥7 h	1078	57·2	2568	56·5	1034	50·9	4680	55·3	
Food item frequencies									
Dark bread									
Once a week or less	391	20·8	847	18·6	436	21·5	1674	19·8	<0·001
2–6 times a week	914	48·5	2440	53·7	952	46·9	4306	50·9	
At least once a day	578	30·7	1259	27·7	644	31·7	2481	29·3	
Milk or buttermilk									
≤4 times a week	263	14·0	615	13·5	302	14·9	1180	13·9	0·247
5–7 times a week	333	17·7	896	19·7	382	18·8	1611	19·0	
Several times a day	1287	68·3	3035	66·8	1348	66·3	5670	67·0	
Cooked vegetables									
Once a week or less	1057	56·1	2562	56·4	1127	55·5	4746	56·1	<0·001
2–6 times a week	604	32·1	1617	35·6	659	32·4	2880	34·0	
At least once a day	222	11·8	367	8·1	246	12·1	835	9·9	
Fresh vegetables									
Once a week or less	284	15·1	745	16·4	379	18·7	1408	16·6	<0·001
2–6 times a week	723	38·4	2040	44·9	855	42·1	3618	42·8	
At least once a day	876	46·5	1761	38·7	798	39·3	3435	40·6	
Fruits or berries									
Once a week or less	430	22·8	939	20·7	391	19·2	1760	20·8	<0·001
2–6 times a week	768	40·8	2151	47·3	935	46·0	3854	45·6	
At least once a day	685	36·4	1456	32·0	706	34·7	2847	33·6	
Fresh juice									
<1 times a week	745	39·6	964	21·2	257	12·6	1966	23·2	<0·001
1–4 times a week	715	38·0	2078	45·7	681	33·5	3474	41·1	
At least 5 times a week	423	22·5	1504	33·1	1094	53·8	3021	35·7	
Salty snacks									
None	460	24·4	239	5·3	70	3·4	769	9·1	<0·001
<1 times a week	1067	56·7	2034	44·7	462	22·7	3563	42·1	
At least once a week	356	18·9	2273	50·0	1500	73·8	4129	48·8	
Pizza									
None	777	41·3	877	19·3	230	11·3	1884	22·3	<0·001
<1 times a week	1006	53·4	3211	70·6	1237	60·9	5454	64·5	
At least once a week	100	5·3	458	10·1	565	27·8	1123	13·3	
Hamburgers or hot dogs									
None	969	51·5	1057	23·3	257	12·6	2283	27·0	<0·001
<1 times a week	867	46·0	3042	66·9	1178	58·0	5087	60·1	
At least once a week	47	2·5	447	9·8	597	29·4	1091	12·9	

Furthermore, the associations of low, medium and high STI with being thin and overweight appear as OR and 95 % CI in Table 4. A low STI was more common in overweight than in normal-weight or thin subjects. Compared with medium STI, high STI subjects exhibited a higher risk for being thin (OR 1·20; 95 % CI 1·02, 1·41) and a lower risk for being overweight (OR 0·79; 95 % CI 0·67, 0·92), indicating an inverse relationship between sugary food consumption and BMI. Low STI subjects exhibited a higher risk for being overweight (OR 1·32; 95 % CI 1·14, 1·53).

**Table 4.** Sweet Treat Index (STI) related to BMI, compared with normal weight, among all subjects (*n* 8461)* (Odds ratios and 95 % confidence intervals)

	BMI groups											
STI	Normal weight		Thin		Comparison, normal weight × thin			Overweight		Comparison, normal × overweight		
	*n*	%	*n*	%	OR	95 % CI	*P*	*n*	%	OR	95 % CI	*P*
Low	1338	21·4	215	22·2	1·05	0·89, 1·25	0·551	330	26·6	1·32	1·14, 1·53	<0·001
Medium	3389	54·2	497	51·3	1			660	53·1	1		
High	1523	24·4	257	26·5	1·20	1·02, 1·41	0·032	252	20·3	0·79	0·67, 0·92	0·003

* Results from multinomial logistic regression. Model adjusted for sex, age, maternal socio-economic status, sleep duration during weekdays and physical activity. The group ‘overweight’ includes obese subjects.

**Fig. 1.**
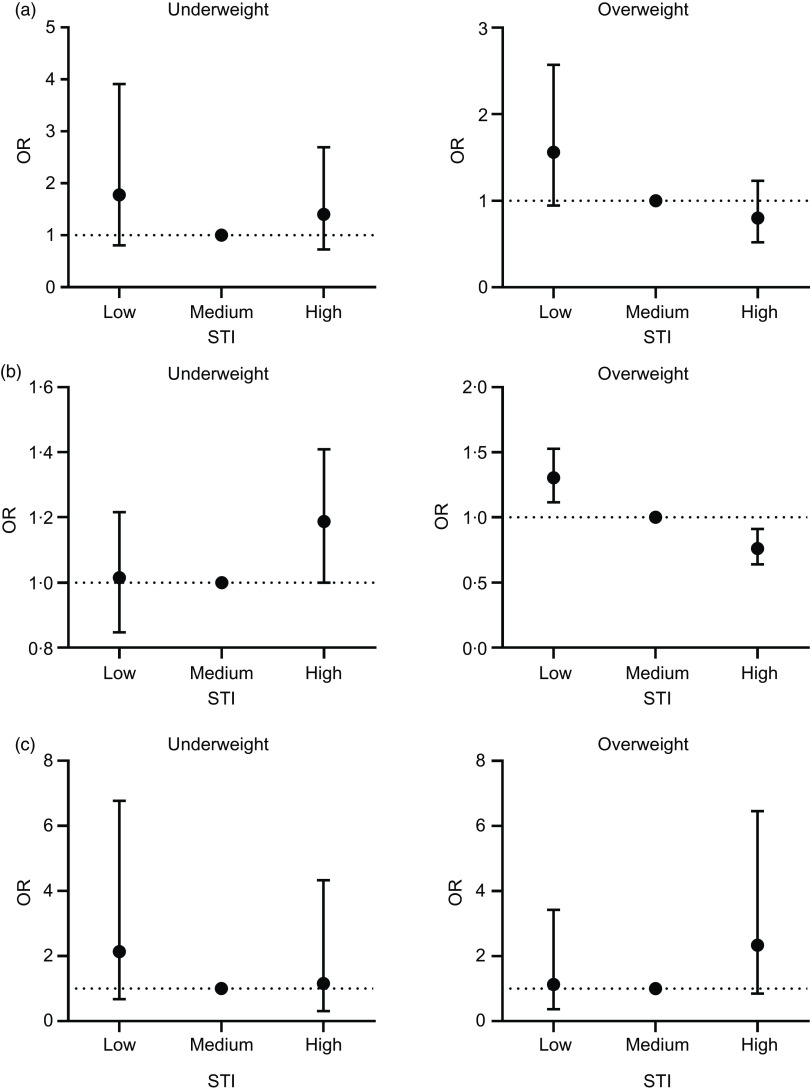
OR and 95 % CI for the Sweet Treat Index (STI) related to the BMI categories, compared with normal weight, stratified by sleep groups (a, b and c). Results from multinomial logistic regression. Model is adjusted for sex, age, maternal socio-economic status and physical activity. The group ‘overweight’ includes obese subjects. (a) Sleep, less than recommended (*n* 689); (b) sleep, recommended (*n* 7606); (c) sleep, more than recommended (*n* 166).

In addition, the risk of being thin and overweight was stratified based on the sleep duration categories, since we found an interaction between STI and sleep duration (*P* = 0·001; Fig. 1). Among children adhering to the recommended amount of sleep, we found results resembling those in the entire sample, although the higher risk of being thin for those in the high STI group was barely significant (95 % CI 1·00, 1·41). Among children sleeping less or more than recommended, we did not find any statistically significant risks for being thin or overweight.

## Discussion

In this study, we observed an inverse relationship between the consumption frequency of selected sugary products and BMI in a large cohort of Finnish preadolescents. Those with a low consumption frequency exhibited a higher risk for being overweight. In contrast, those with a high consumption frequency had a higher risk for being thin and appeared at lower risk for being overweight. We utilised data from the well-defined Finnish Health in Teens cohort, which includes several self-reported lifestyle factors, including sugary product consumption, sleep duration and PA levels, among over 8000 Finnish children aged 9–12 years.

For the study purposes, we created an index to measure the weekly frequency of sugary product consumption and investigated its relationship to being both overweight and thin. Indices combining a variety of sugary foods and drinks have been used in several studies^(34–36)^. To the best of our knowledge, ours represents the first study to investigate the association between frequency of consumption based on a sum score of selected sugary products and BMI. In an Italian study with close to 60 000 11–15-year-old subjects, the more frequent consumption of ‘unhealthy foods’ correlated with a lower risk of being overweight among all subjects except 11-year-old girls^(37)^. In that study, unhealthy foods encompassed sweets, soft drinks and crisps, which were combined to create a summed variable. Their index did not include purely sugary foods; nevertheless, their results are similar to ours.

Moreover, previous studies assessed the relationships of the consumption frequencies of individual sugar-rich food items and BMI in children and adolescents worldwide^(38–40)^. The results from the large, cross-national HBSC study among 137 593 participants across thirty-four mostly European countries support our findings, whereby a higher consumption frequency of sweets (defined as candy and chocolate) was associated with a lower OR for being overweight in thirty-one of thirty-four countries^(39)^. In addition, a study among 2906 Saudi Arabian adolescents aged 14–19 years with less frequent sugary drink consumption showed a higher risk of being overweight and obese compared with more frequent consumption; moreover, overweight subjects consumed sweets less frequently than normal-weight subjects^(38)^. In a Norwegian study among 2281 children aged 6–16 years, less frequent consumption of sweets was associated with obesity^(40)^.

Several possible explanations exist for our findings. Given the absence of information about portion sizes, we cannot exclude the possibility that children who consumed sugary products less frequently were actually consuming larger portions than children who consumed them more often, thereby resulting in higher energy intake. Furthermore, a child’s consumption frequency of sugary products may be restricted by parents through their attempts to promote healthy eating^(41)^. Controlling and limiting might lead children to eat sugary products in large quantities once the restriction is lifted. In 3–7-year-old children, restricting eating highly palatable foods, such as sweet foods, resulted in a higher intake of those foods following the removal of restrictions^(42–44)^.

Another reason for limiting the consumption frequency of sugary products may result from parental efforts to restrict eating in children already considered overweight. Conflicting evidence exists of the association between maternal feeding practices and a child’s later BMI. Specifically, in one study, maternal restriction of a child’s eating at 3–5 years was associated with a higher BMI 2 years later^(45)^, while another study found that only after gaining weight did maternal feeding practices become restrictive^(46)^. Moreover, maternal use of restriction promoted eating in the absence of hunger^(41)^. However, these results focus on children younger than those in our sample. Among older children, parental restriction may no longer prove effective, since adolescents enjoy more freedom to buy snacks and beverages on their own. However, conflicting results have been reported for 12–17-year-old Dutch adolescents, whereby perceived restrictive parenting practices correlated with less soft drink consumption^(47)^. The association between being thin and a high consumption frequency may be explained by parents encouraging or not restricting thin children to eat sugary foods more often in order to gain weight^(48)^.

Contrary to our results, previous studies among 2–19-year-old children and adolescents in Saudi Arabia and Spain found that the frequent consumption of sugary products, including sweets, soft drinks and chocolate, was associated with excess weight^(49,50)^. More frequent consumption of sweets and soft drinks correlated with obesity in 2–14-year-old Spanish children^(50)^. That sample population, however, was recruited during healthcare visits and was also smaller (*n* 1188) than ours, with a wider age range than in our study, possibly explaining the contradicting results. Moreover, information was reported by parents. Among Saudi Arabian school girls aged 12–19 years, a higher consumption frequency of chocolate and sweets was associated with obesity^(49)^. Again, explanatory factors accounting for these conflicting results may stem from the different age ranges and the smaller sample size (*n* 512). Furthermore, sex and cultural differences may account for the differing results^(51,52)^.

Along with sugary food items, the self-administered FFQ contained nine other food items. We found that the highest proportion of children consuming fast foods and salty snacks was among those with a high STI, indicating an overall low-quality dietary pattern among them. Frequent consumers of sweet foods were also frequent consumers of high-fat foods such as crisps in a study of 1-year-old children^(53)^. Our results do not support the speculation that the high consumption of sweets associates with eating less fatty foods and, thus, leads to relatively low total energy intake^(39)^.

We did not find a significant association between STI and BMI among those children who slept less than recommended. However, subjects with a high STI were overrepresented in this subgroup. In previous studies, a shorter sleep duration was linked to unhealthy eating habits and more frequent consumption of sugary products such as sweets, chocolate and cakes among school-aged children^(26,54–56)^. A short sleep duration was associated with an increased BMI in adolescents^(57)^, and our overall findings indicate that the weight status differs based on sleep duration. When we stratified our analysis by sleep, we found a similar relationship between the STI and BMI groups in those who slept the recommended amount as in the whole sample. This was not surprising as they constituted the majority of the sample. However, no associations were observed in those sleeping less or more than recommended. This could be due to small sample sizes.

Among Finnish children, the prevalence of overweight and obesity in this age group has reported to be similar^(58)^ to or higher^(59)^ than that presented here. The corresponding result, with the combined prevalence of 15·4 % in fifth grade children, was reported in ten school health units during 2007–2008^(58)^, while more recent data from the national THL Avohilmo register from 2014–2015 indicated that roughly 28 % of children aged 7–12 years were at least overweight^(59)^. Regarding the maternal SES at the birth of the child, our sample seems to have slightly different SES compared with data from Statistics Finland concerning all 25–34-year-old women living in Finland in 2010^(60)^. We marked an overrepresentation of upper-level employees (30 *v*. 18 %) among our mothers, while the proportions of lower-level employees and manual workers among our sample resemble those in the young female population (39 *v*. 42 % and 12 *v*. 15 %, respectively). A reason for the overrepresentation of upper-level employees could be due to our sampling which concentrated on densely populated, mostly urban-like areas and lacked subjects living in the most rural, scarcely populated areas. As the maternal SES was associated with both weight status and the STI of the child, it was included in the analysis as a confounder; thus, its effect on the relationship between the STI and weight status has been taken into account. Our findings can be generalised to European populations with high maternal SES living in urban-like areas.

The FFQ we used to assess habitual food choices consisted of sixteen food items. We chose to use a short FFQ as it is easy and less burdensome to complete as a part of a longer survey. Due to the shortness of the FFQ, we were not able to estimate consumption of all possible sugar sources; thus, the STI focused on foods and drinks that are generally consumed as sweet treats. The subjects reported their food choices themselves, which appears more accurate than parental reporting^(61)^. Children are cognitively skilled to self-report from the age of 8 years^(62)^, and as children consume a part of their food outside homes, out of the reach of parental supervision, they are likely more capable to report their food choices than their parents.

The strengths of the present study include its large population-based, geographically diverse sample relying on recent data from the 2010s, and the reliable anthropometric measurements completed by trained fieldworkers. Furthermore, we investigated the associations of consumption frequency not only with being thin and but also with being overweight. Being thin is relatively common in this age group, and including thin children as a separate group helps to identify their specific consumption behaviours and related risks. Moreover, by using an index that covers a group of sugar-rich foods and drinks, we could characterise a dimension of subjects’ eating behaviours instead of the consumption of individual food items alone^(34)^. Our inability to assess all possible sources of dietary sugars, as discussed above, is a limitation. Moreover, the lack of information regarding portion sizes also represents a limitation; nevertheless, a FFQ without information on quantities are considered a fast and useful way to rank subjects^(23)^. Under- and overreporting remain a possibility and, thus, represent a further limitation. Underreporting is of concern in dietary questionnaires, particularly regarding foods considered unhealthy and in studies involving obese subjects^(63,64)^. Overreporting, on the other hand, might be a problem among underweight individuals^(64)^. However, in the International Study of Childhood Obesity, Lifestyle and the Environment validation study among 11–12-year-old children, the validity of the FFQ was independent of BMI^(25)^. Moreover, the tendency to misreport might increase with age^(62)^. In general, FFQ may overestimate consumption frequencies^(23,24)^. Due to the lack of information on energy intake, we were not able to estimate the level of misreporting. However, frequent consumption of sweet treats might affect body weight by suppressing appetite without contributing to total energy intake^(65)^. We used the International Obesity Task Force’s BMI classification to define weight status, which may overestimate the prevalence of thinness, and on the other hand, underestimate the prevalence of overweight and obesity when compared with the WHO classification^(66)^. Implications of using different reference systems should be investigated in further studies. The possibility of selection bias due to the low response rate cannot be dismissed. It might have been possible to increase the response rate by sending reminders. However, this would probably not have contributed to the heterogeneity of the sample and thus would have had only minor impact on our conclusions^(67,68)^. Occupation as the indicator of the maternal SES was obtained at the time of child’s birth, which might not reflect the actual situation at the time of data collection and could be considered as a limitation in this study. Finally, a further limitation is the cross-sectional study design where both the outcome and the exposure were measured at the same time, which means that no conclusions regarding causation can be made. Thus, our findings should be confirmed through longitudinal studies with repeated measures of both exposure and outcome.

In conclusion, the frequent consumption of sugary products diminished the risk of being overweight and concurrently increased the risk of being thin among preadolescents. Focusing solely on the consumption frequency of sugar-containing products is reasonable from the viewpoint of dental health, but in dietary counselling and health promotion, it might prove misleading. A whole-diet approach together with portion size should be highlighted.
